# Analytical tool for optimization of position sensors based on eddy currents effect

**DOI:** 10.1016/j.heliyon.2022.e11920

**Published:** 2022-11-26

**Authors:** Andreia Faria, Luís Marques, Luís Vale, Carlos Ferreira, Filipe Alves, Jorge Cabral

**Affiliations:** a ALGORITMI Center—University of Minho, Guimarães, Portugal; b Centre of Physics of Minho and Porto Universities, Braga, Portugal; c CEiiA–Centro de Engenharia e Desenvolvimento, Porto Portugal; d Integrated Micro and Nanotechnologies, International Iberian Nanotechnology Laboratory, Braga Portugal

**Keywords:** Planar coil, Mutual-inductance, Analytical tool, Eddy current, Finite element method, Target

## Abstract

This paper proposes an open-source tool for a fast and precise calculation of the inductance of planar coils in the presence of conductive targets. This tool, based on Grover equations, is versatile to any coil and target geometries. The performance of this tool has been validated by comparing the analytical results with experimental data and Finite Element Model (FEM) simulations. When compared with the experimental measurements, the developed analytical model and the FEM simulation retrieved similar results, with differences below 4%. The proposed tool, in addition to requiring less computing resources, is significantly faster than FEM simulations. This shows the effectiveness of the proposed approach for the analytical calculation of the inductance of planar coils in the presence of conductive plates. It combines accuracy, versatility, speed, and no need for high computational resources.

## 1 Introduction

In recent years, novel sensors based eddy current effect have been developed [1, 2, 3, 4, 5] and exploited in different applications in health [6, 7], industry [8], aerospace [9] and automotive [10] areas.

Position sensors based on eddy currents, in order to become more competitive, usually use coils printed on PCB. The use of this type of coils makes it possible not only to lower the production and assembly costs but also the manufacturing errors of the coils and of the sensor's calibration. However, during the sensor development, its design phase can be very time-consuming. Due to the complexity of planar coil inductance calculations in the presence of a metallic target, two approaches are commonly used at this stage. Initially, finite element simulations are made to ensure that the combination of the coils with the different target positions meets the requirements defined for the sensor. After obtaining the desired values, the simulation models are validated via experimental measurements. If the simulation model used by the FEM simulations is well configured, generally, the results obtained are very close to those obtained experimentally. Based on the experience of the authors, errors are usually below 10%. Despite this performance advantage, FEM simulations have the disadvantage of requiring a long running times to generate the solutions. The more refined and complex model, the longer it takes to render a solution. Another drawback is the fact that they use dedicated software, which is expensive and requires a lot of computing resources.

Considering the growing interest in sensors based on eddy currents, and the limitation of the approach currently used during the design phase, this work proposes a tool to optimize this process. An approximate analytical model to calculate the inductance resulting from planar coils in the presence of a metallic target is presented. Since this model is analytical, it can quickly and economically obtain results with similar errors to those of FEM models. Section 2 describes the developed model, the method used to do the inductance calculation of planar coils, and the approximation used for the integration of the target in inductance calculations. The validation of the developed model was performed by comparing it with FEM simulations and with experimental measurements for the sensor use-case. Section 3 describes the architecture and working principle of the selected sensor. Section 4 shows how the developed model was applied to the chosen sensor. Also, the FEM model created and the measurement setup used are detailed. The analysis and comparison of the results obtained through the three methodologies are described in Section 5. The main conclusions reached and possible improvements to be implemented in the developed model are presented in Section 6.

## 2 Analytical model

The inductance of a system composed of a coil in the presence of a metallic target is the result of the influence of the magnetic field generated by the metallic target on the inductance of the coil. Eddy currents induced on the surface of the metallic target create a magnetic field that reduces the original magnetic field of the coil. Using the equivalent circuit of the coil and target system the total inductance, neglecting the coils and target resistance, can be estimated by:

(1) $$L_{\text{Total}}{=L}_{\text{Coil}}-M_{\text{Coil}+\text{Target}}^{2}{/L}_{\text{Target}}$$

Thus, the process of calculating the resulting inductance of a planar coil in the presence of a metallic target can be divided into three phases. One focused on the calculation of the self-inductance value of the coil ($L_{\text{Coil}}$), another on the self-inductance of the metallic target ($L_{\text{Target}}$), and lastly on the calculation of the mutual inductance between the coil and the metallic target (M_Coil+Target_).

Regarding the calculation of the self-inductance of a planar coil, the model presented in [11] is used. This is an analytical model based on the Grover equations to calculate the self and mutual inductance of and between planar coils. This method considers a planar coil as a set of straight segments. Thus, the coil's inductance is the result of the sum of the self-inductances of all its segments, plus the mutual inductance between them.

Grover equations are considered the most accurate, and the developed model presented results in agreement with those obtained by the commonly used methods, with the advantage of being faster and more versatile.

**Figure 1:**
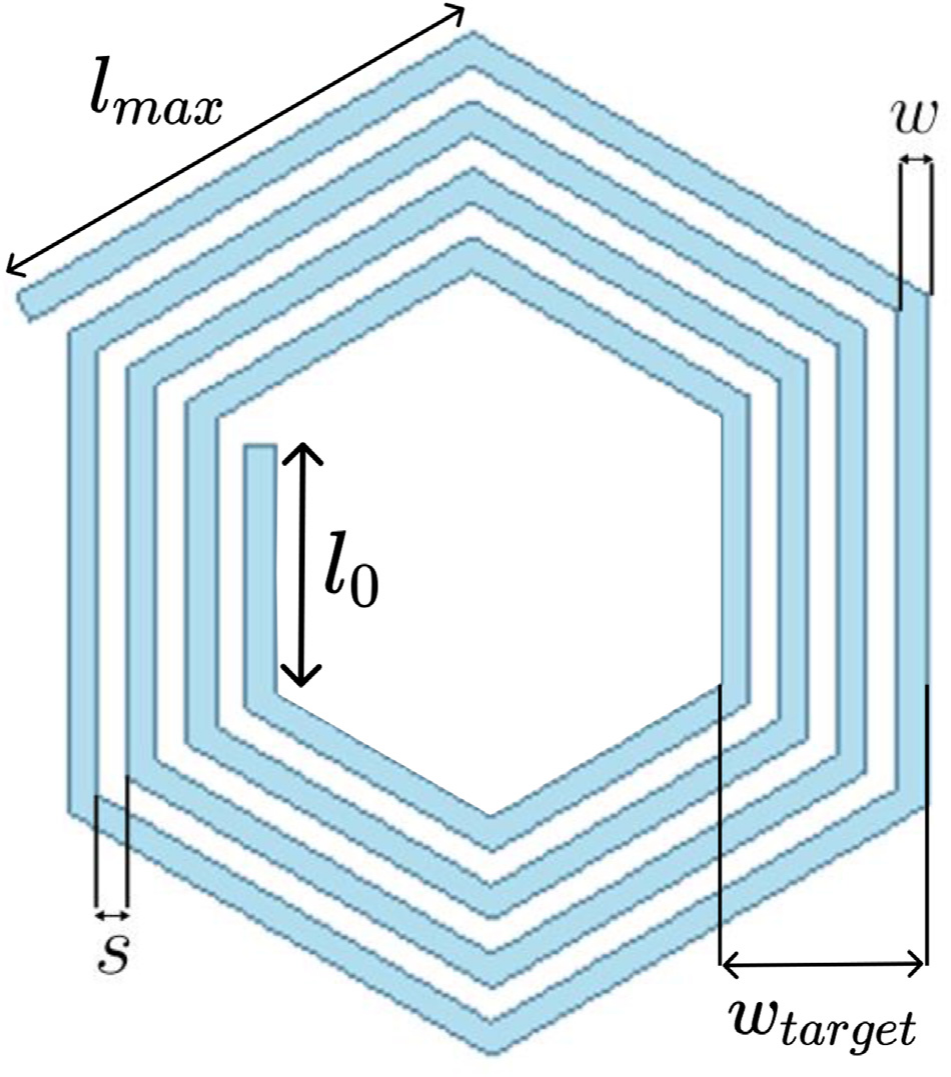
Hexagonal coil and the corresponding target geometry which is considered in the analytical model to calculate the inductance between them.

For the calculation of the target's self and mutual inductance, the Grover equations will also be used as a basis. In this calculation some approximations were assumed, concerning the spatial distribution of eddy currents on the target: 1) The eddy currents are induced in the area under the coil, being negligible outside the overlapping area between the coil and target, as done by HiroyukiWakiwaka et al [12] and Norhisam Misron et al [13]; 2) For inductance calculation purposes, the eddy currents distribution is approximated by an one-turn coil, with a track width equivalent to the space between the inner and outer edges of the coil [12]. Figure 1 shows an example of a hexagonal coil with 4 turns and in shaded blue the shape of the target that is considered by the analytical model.

It is noticeable that the outer and inner sides of the coil are responsible for defining the shape and width of the one-turn coil that is used to represent the target.

**Figure 2:**
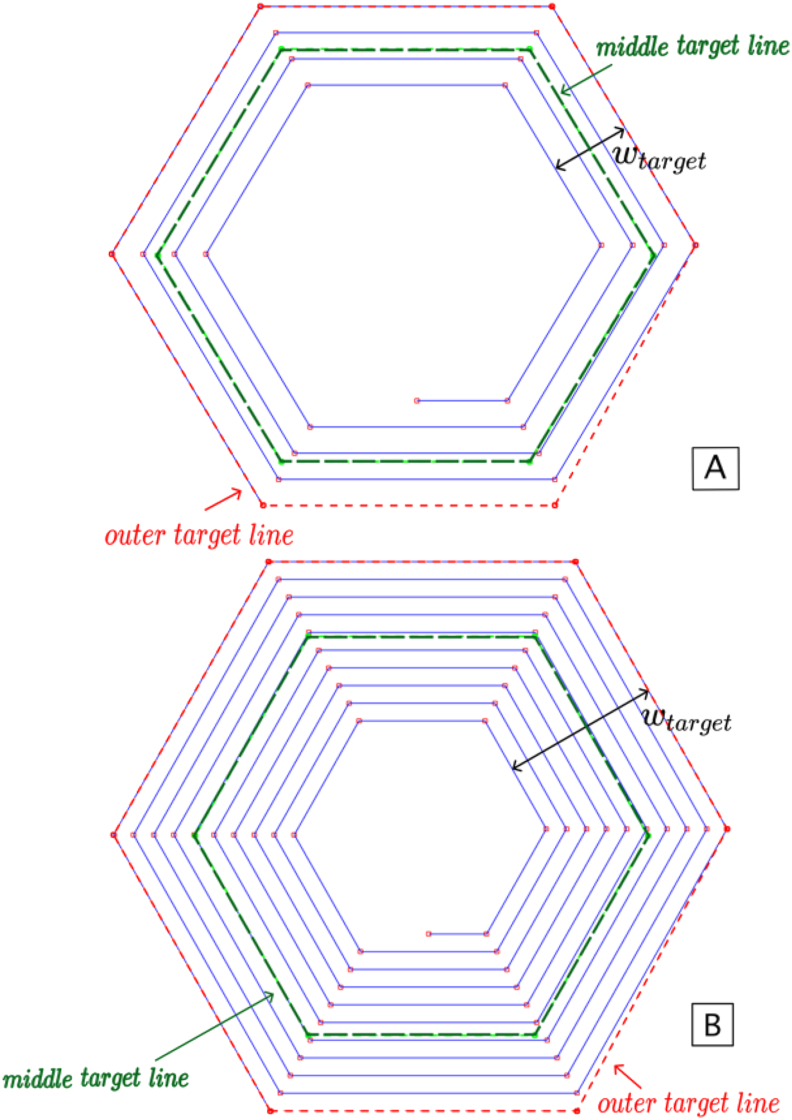
Hexagonal coil and the corresponding target geometry which is considered in the analytical model to calculate the inductance between them (A: Coil with 4 turns; B: Coil with 10 turns).

For the calculations of the self-inductance of the target (L_Target_), and the mutual inductance between the target and the coil (M_Coil+Target_), the middle position of the one-turn coil filament was used. This corresponds to the middle point between the inner and outer edges of the coil, as marked in green dashed lines in Figure 2. This figure shows two examples of how the analytical model considers a hexagonal coil and its target in the inductance calculations, for 4 and 10 turns. In blue are represented the filaments of the hexagonal coils and in green are those of the coil that represents the target.

In order to obtain the target dimensions, as the coils have regular geometries, the radius of the circle containing the outermost filament of the coil was calculated. Later, this radius is used to calculate the length of the sides of the target and enabling the calculation for the coordinates of each of the target vertices. Thus, the target parametrization for the position in which it is completely over the coil is obtained. However, if it is only partially overlapping the coil, it is necessary to intersect its areas in order to obtain the overlapping area. Figure 5 exemplifies the translation of a target over an octagonal coil and how this movement is reflected in the target's geometry. The resulting geometry is the one that will be considered later in the calculations of the mutual inductance between the target and the coil.

Once the target geometry is defined, the inductance of the coil overlapped by a target can be calculated.

Due to the target's approximation to an one-turn coil, its self-inductance and mutual inductance with the coil are calculated using the same method used for the 2-layer planar coil.

To extract the total inductance of the coil, as proposed in [11], first it has to be calculated the self-inductances of the 1-layer coil (L_Coil_) and the target one-turn coil (L_Target_), as well as the mutual inductance between both (M_Coil+Target_) (according to eq. (1)).

There are other research approaches to calculate (analytically) the inductance of planar coils in the presence of eddy currents through the magnetic fields [14]. These approaches provide similar results, but the analytical solution is more complex requiring higher processing capabilities.

## 3 Differential coils angular position sensor

The proposed platform has been developed in the scope of a research project that targets the development of a Differential Coils Angular Position Sensor (APS). This sensor has the function of measuring angular displacements based on the eddy current effect, using planar coils printed on PCB and conductive plates, both strategically designed and placed to measure a range of 90°, with a maximum error of 1°.

**Figure 3:**
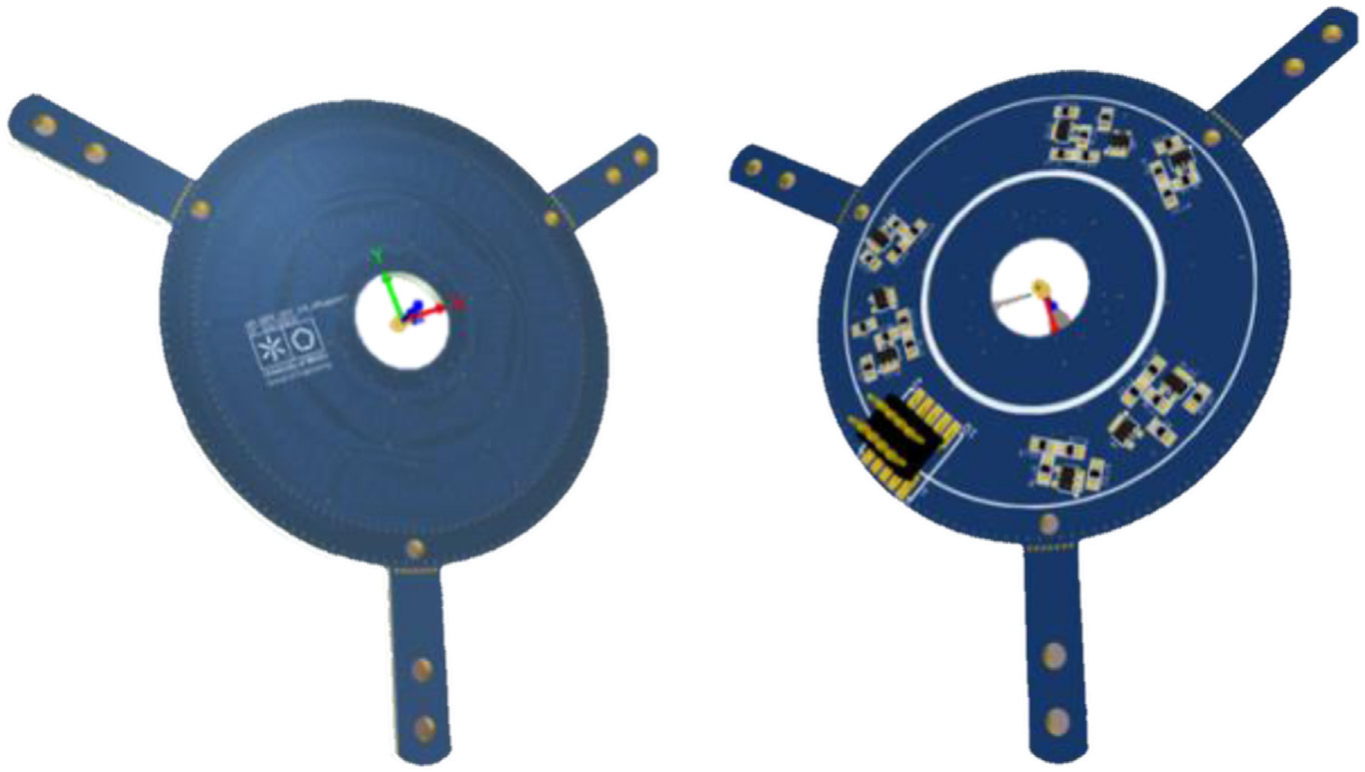
Differential coils APS–PCB sensor.

**Figure 4:**
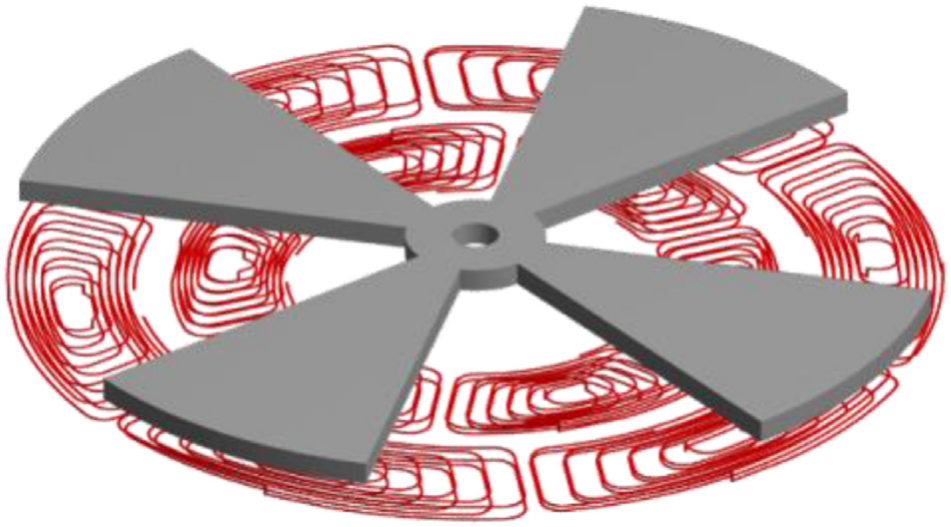
Differential coils APS–coils and target geometry.

This sensor has numerous applications. It can be applied, for example, to control the angular position of a vehicle's auxiliary braking motor. To enable measurement of the motor's angle of rotation with this sensor, it is necessary to couple on the motor shaft a conductive plate (shown in Figure 4) and the PCB board with the signal conditioning circuit, as depicted by Figure 3.

**Figure 5:**
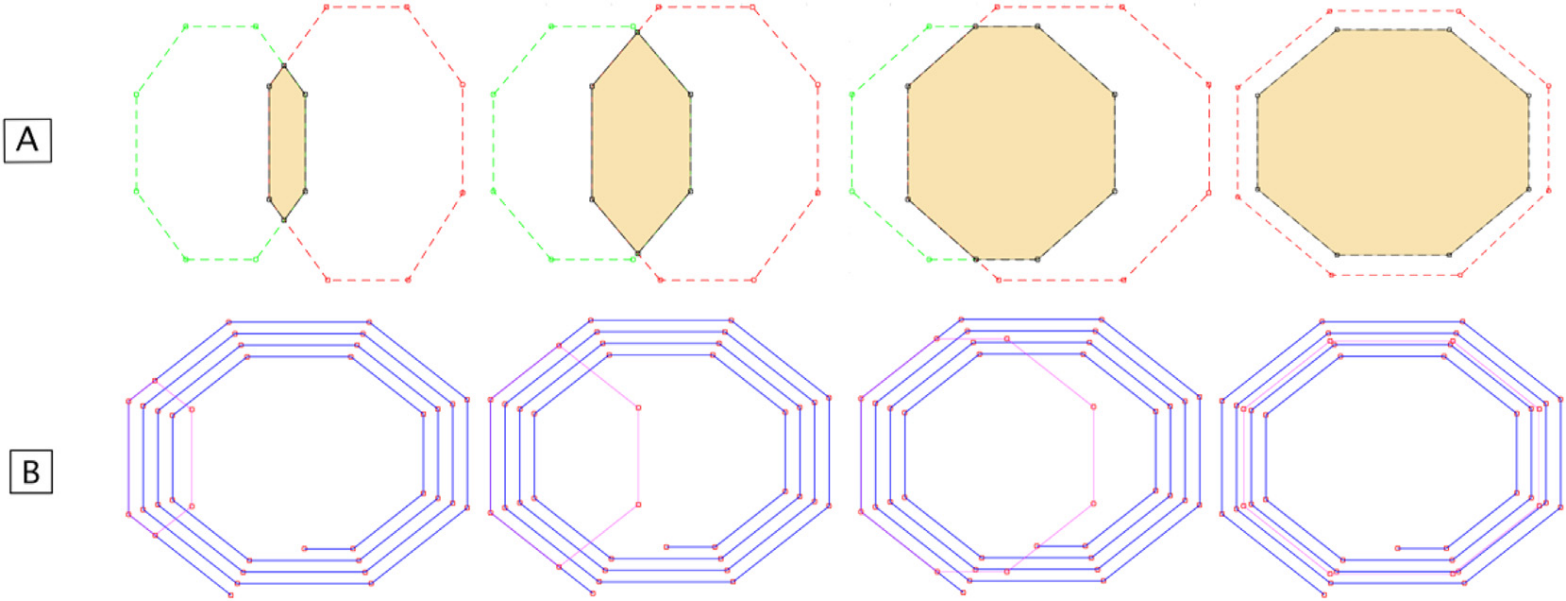
Target moving and the resulting geometry (A: Sequence with middle coil line; B: Sequence with coil filaments).

This sensor features a particular design and positioning of its coils. This design was the result of an intensive research work in order to achieve the best combination between the geometry of the coils, their interconnection, and positioning, so that the sensor would offer the best performance in terms of resolution and precision.

**Figure 6:**
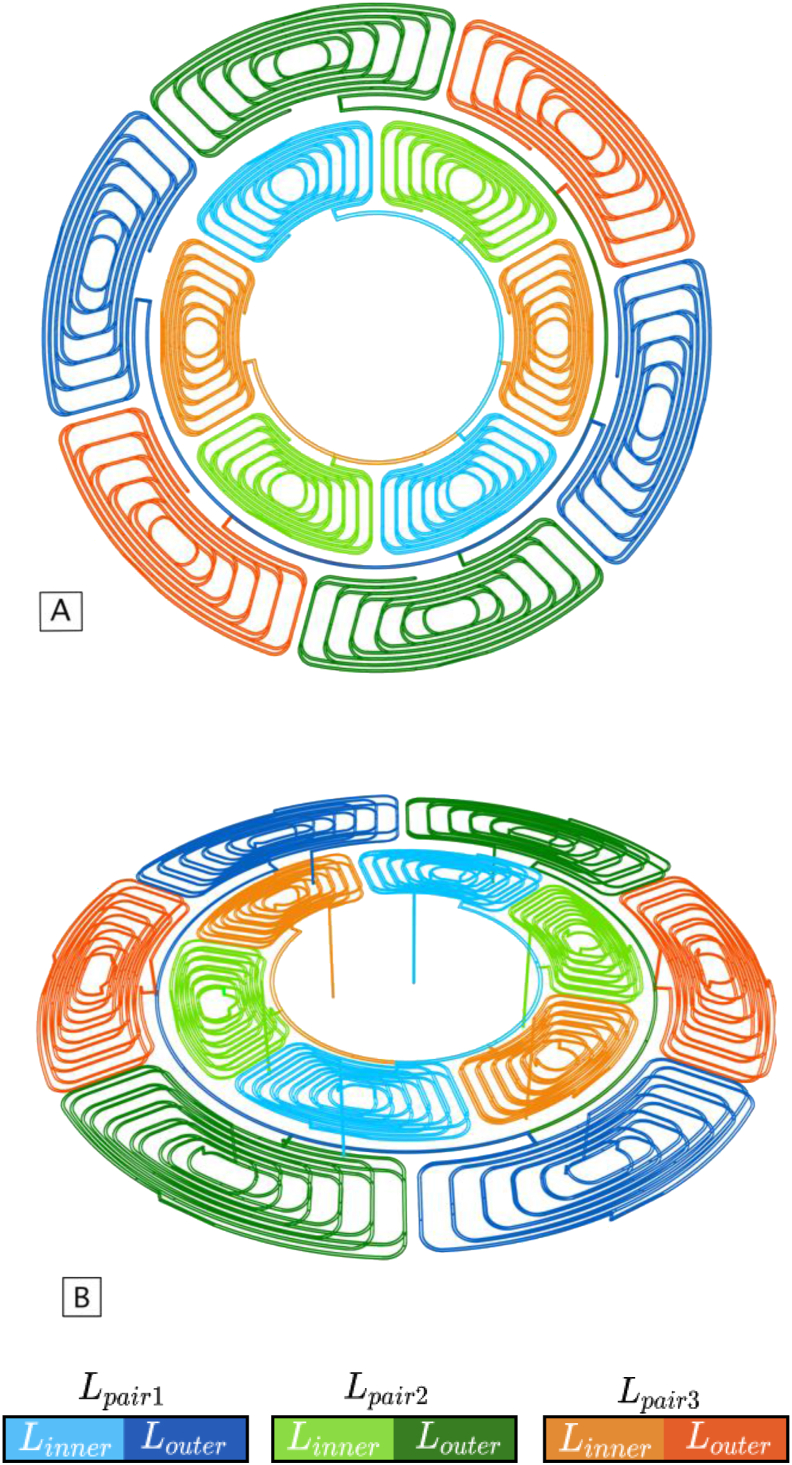
Sensor coils design. A: top view; B: perspective view.

Figure 6A and B shows, in more detail, the coils architecture used in the proposed sensor.

It is noticeable that there are six equal coils in an inner radius (illustrated in a lighter tone) and another six in an outer radius (illustrated in a darker tone).

The coils are connected in pairs with 180° displacement, originating a total of three pairs of coils in each inner and outer radius.

In order to better understand this configuration, in Figure 6A each pair of coils in the inner and outer radius coils are highlighted in the same color, having the inner coil a lighter tone, and the outer one a darker tone. In Figure 6B, it can be seen that all the coils were designed to have two layers, which enabled to increase the inductance of each coil, and the associated magnetic field, without increasing its total size.

Based on the sensor operation, the inductance value of each pair of coils differs according to the overlapping area between the conductive target and the coil, due to the presence of eddy currents. These create a second, weaker and opposing alternating magnetic field, as described in Lenz’ Law. The sum of both magnetic field results in a weaker magnetic field and, also, a smaller coil inductance. In sum, the larger the overlapping area, the greater the amount of eddy currents generated and smaller is the resulting coil inductance.

In order to measure/quantify this inductance variation in the designed coils, this sensor uses a simple LC oscillator for each pair of coils. The output of each oscillator is a square wave, whose frequency is dependent on the inductance of its own pair of coils. As, the spatial magnetic field modulation is done by the conductive target, as previously described, this is reflected on the inductance of the coils. Resulting in a variation of the frequency of the oscillator. This frequency value can be approximated to Eq. (2).

(2) $$f_{osc}\approx {\left(2\pi \sqrt{LC})\right)}^{-1}$$

where L corresponds to the inductance of a pair of coils and C to the capacitance value of the capacitors of the oscillator.

**Figure 7:**
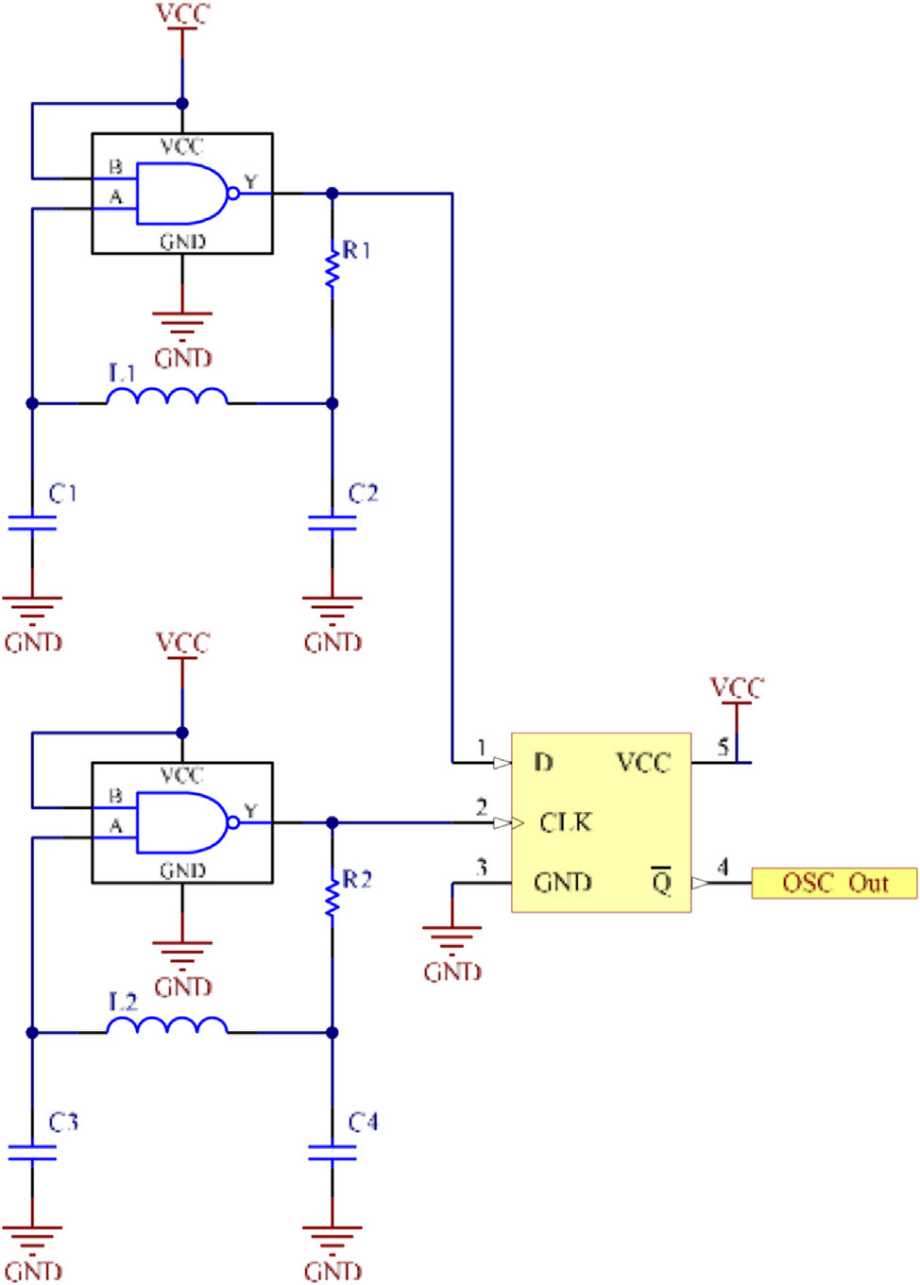
Electronic schematic of LC oscillators and the D-type flip-flop frequency sub-tractor (sliding window).

The differential measurement principle is obtained using a D-type flip-flop (Figure 7). It receives at its inputs the frequency signals of both, inner and outer, oscillators. Thus, the flip-flop output will be the result of subtraction of both signals, in a sliding window manner, $f_{output}=f_{oscInner}-f_{oscOuter}$, that corresponds to a square wave with its frequency varying in a sinusoidal manner with the angular displacement.

Through the analysis of Figure 6A it is perceptible that both inner and outer coils are misaligned. The placement of the coils in the available space was made considering the latter. If the corresponding inner and outer coil pairs were aligned, at the moment when the target would covering the maximum amount of area of the coils, the output wave would have a very reduced amplitude. So, in order to maximize the output amplitude, a 45° rotation of the inner coils was used in relation to the outer ones. Thus, an output signal is obtained with the maximum amplitude, since when an outer coil pair has its maximum inductance value, the corresponding inner pair will have the minimum value. In turn, maximizing the signal amplitude allows the sensor's output signal to have a higher resolution, which is always an asset.

**Figure 8:**
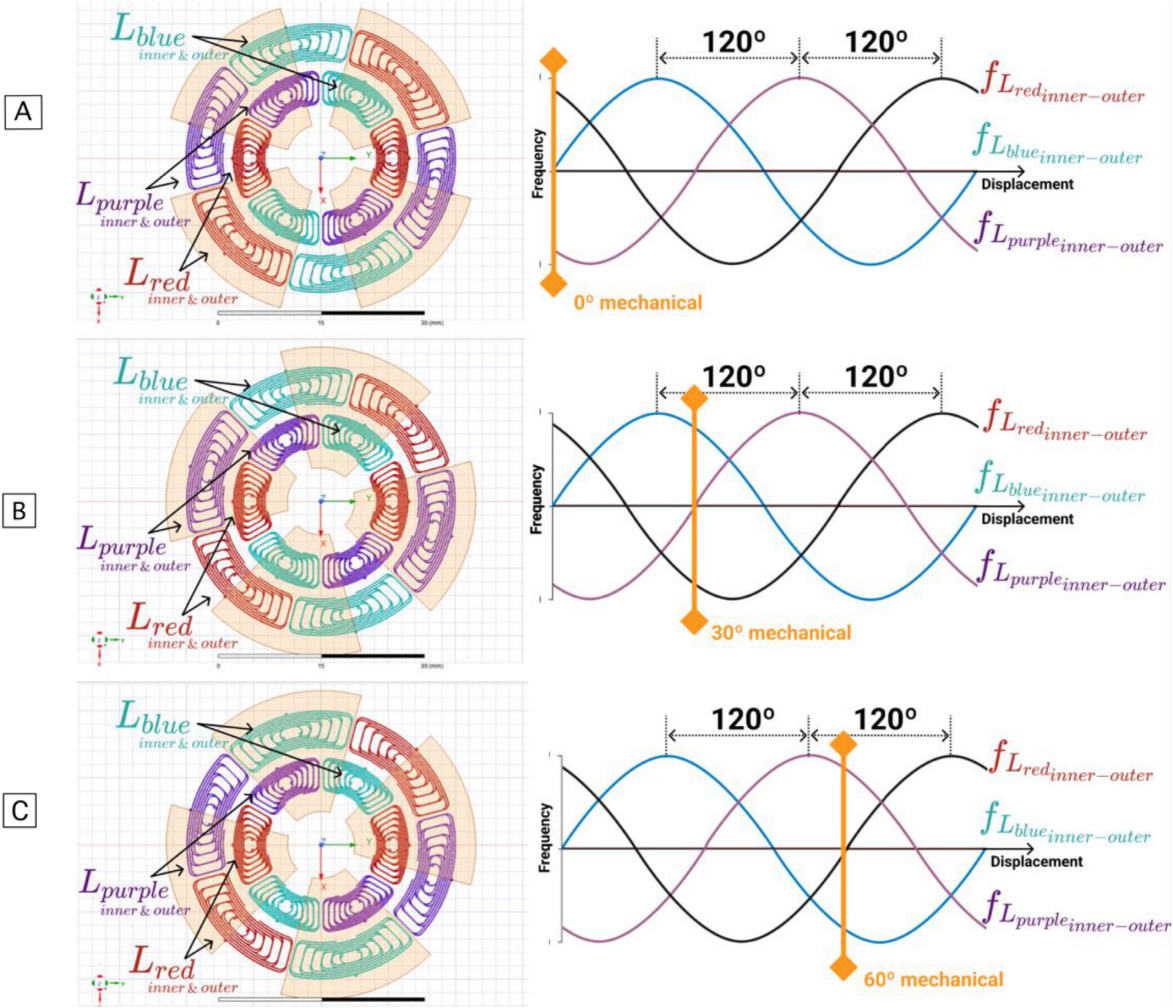
Inductance variations from each differential pair of coils, for a 90° mechanical rotation of the Differential Coils APS (amplitude and phase corrected by software). (A: 0° mechanical angle; B: 30° mechanical angle; C: 60° mechanical angle).

Figure 8 shows the APS sensor's operating mode described before. Its three differential pairs of coils, and how its outputs change with the rotation of the target plates. In a target rotation of 90 mechanical degrees, the frequency signal from each differential pair of coils, f_L_red(inner-outer)_, f_L_blue(inner-outer)_, and f_L_purple(inner-outer)_ (shifted by 120 electrical degrees), does one period (which represents 360 electrical degrees). To interpret the three output signals and extract the angular position of the target, a signal conditioning is done first. The average value is removed from each signal and all amplitudes equalized. Then, the Clarke Transformation is applied, and the angular position obtained.

## 4 Implementation of the methodologies to the angular position sensor

**Figure 9:**
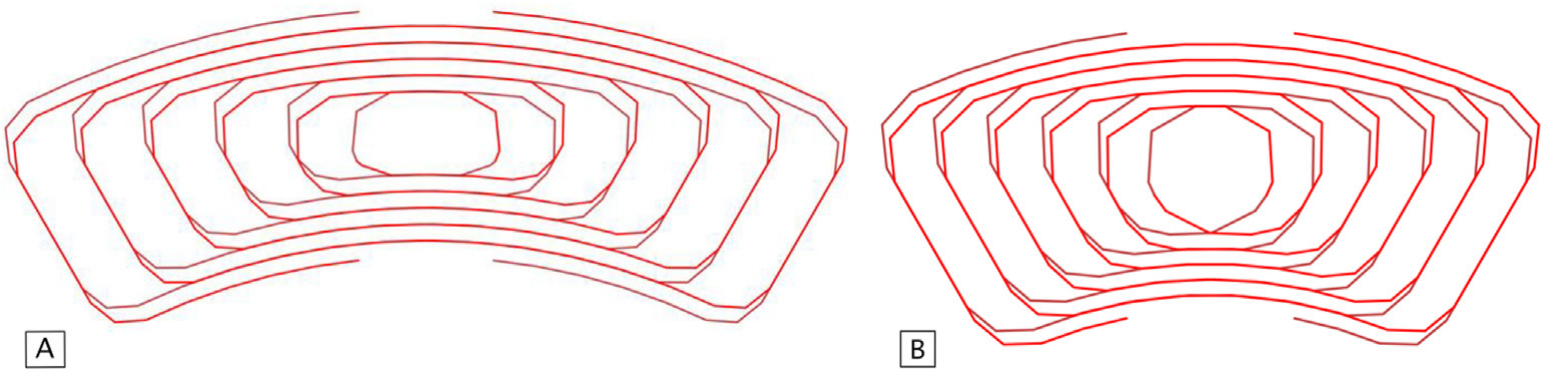
Segments of the outer (A) and inner (B) coils geometries, using a minimum edge length of 1 mm in the discretization process.

As detailed in [11], the analytical method used here is based on straight filaments, and in their central position in the wire. It is possible to observe in Figure 6 that the coils used by the sensor are curvilinear. Therefore, a discretization of the coil into segments was made to enable the use of the calculation tool. Figure 9 shows the filaments used for the inner and outer coils.

The Differential Coils APS is composed by three identical pairs of inner and outer coils, two ground planes (GND) under the coils (at a distance of 0.7 mm and 1.105 mm from the lower coil's layer), and a target over them (at 1 mm distance from the upper coil's layer). However, due to its symmetry, it can be considered in the model only a pair of inner and outer coils.

As mentioned before, to calculate the resultant inductance of the planar coils of the sensor, the GND layers and the target are approximated to a one-turn single coil. To optimize this approximation, a correction factor ($factor_{c}$) was added to Eq. (1), obtaining Eq. (3).

(3) $$L_{Total}=L_{Coil}-M_{Coil+Target}^{2}/L_{Target}\times factor_{c}$$

**Figure 10:**
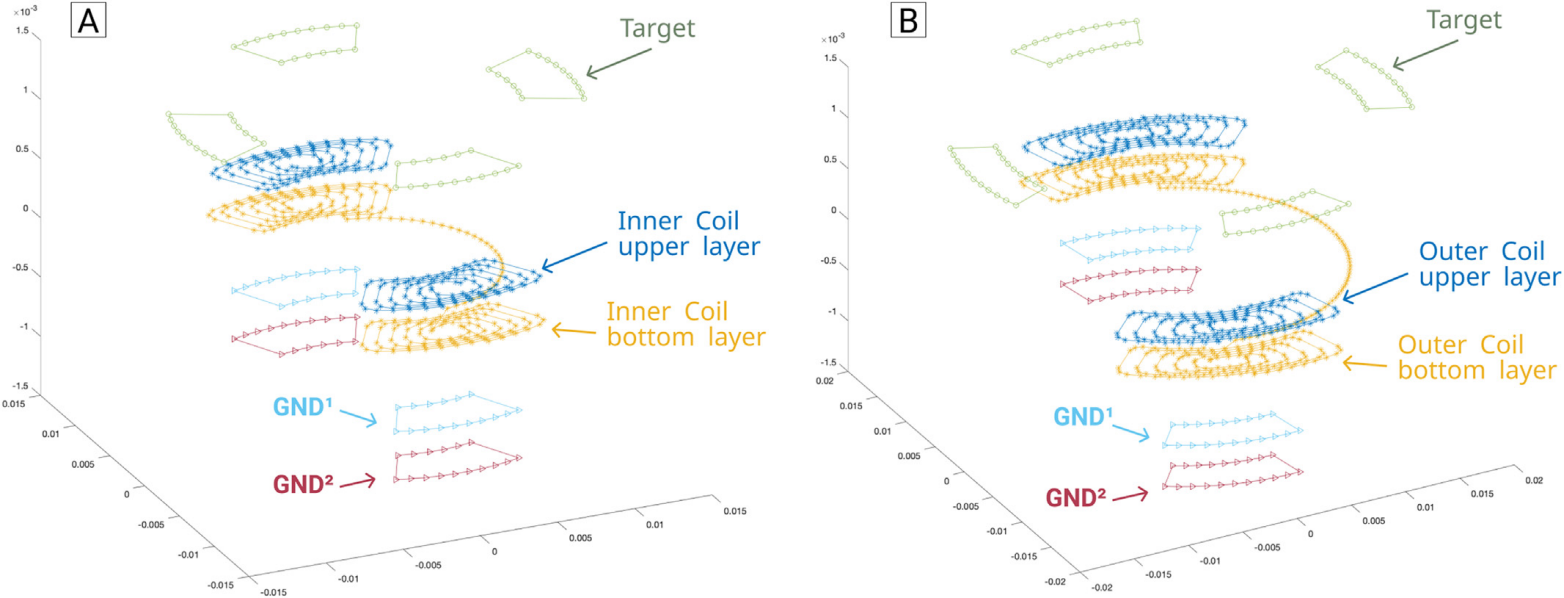
Filaments used by the developed tool to calculate the inductance resultant in the inner (A) and outer (B) pair of coils, in the presence the target and two ground planes.

Regarding the distribution of eddy current density, for the case of GND layers, a correction factor of 30% was considered. In the case of the target, the compensation factor was not used, as its physical dimensions limit the current distribution, unlike the case of the GND layers. Figure 10 shows the sensor's model used in the developed tool.

To evaluate the performance of the proposed tool, a FEM simulation model was also implemented. The Ansys Electronic Software was used, specifically the eddy current mode. The sensor model was simulated using an air-box with an 6 mm relative offset, since there is a saturation of results for offsets higher than that. A test current of 500 mA was applied in the coil's terminals, with a solver frequency of 1 MHz (to match the experimental test frequency). The adaptive setup was configured with an error of 1%, and a minimum of two convergence steps.

As FEM simulations are very time consuming to perform this validation, instead of simulating the pair of coils, it was just simulated one inner and outer coils.

**Figure 11:**
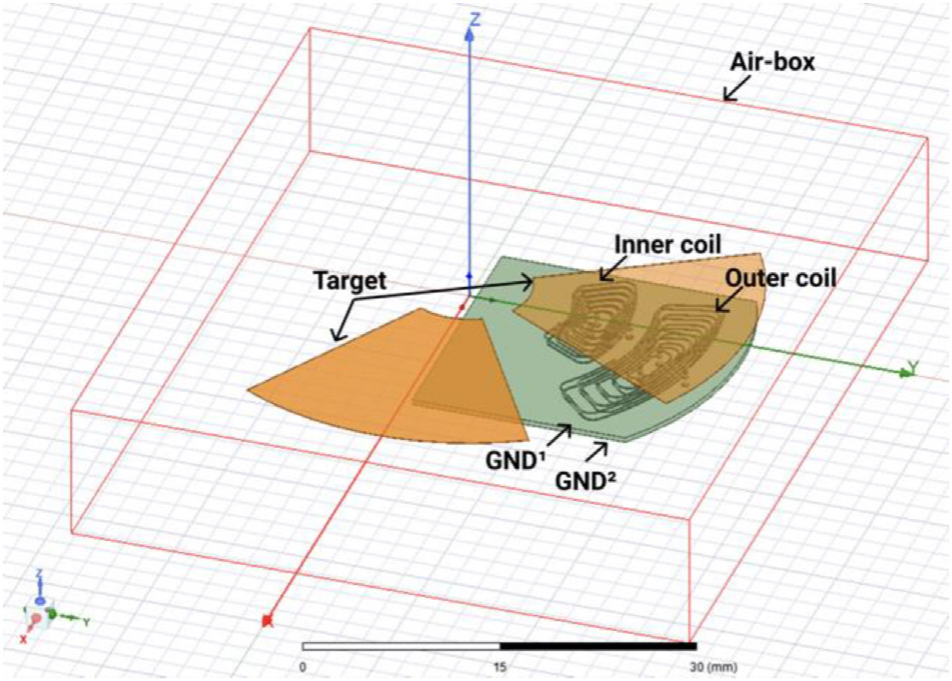
FEM model simulated for validating the inductance values of the pairs of coils of the APS.

By multiplying by two the inductance obtained for each one, the complete inductance of each pair is obtained. Figure 11 shows the FEM model simulated.

**Figure 12:**
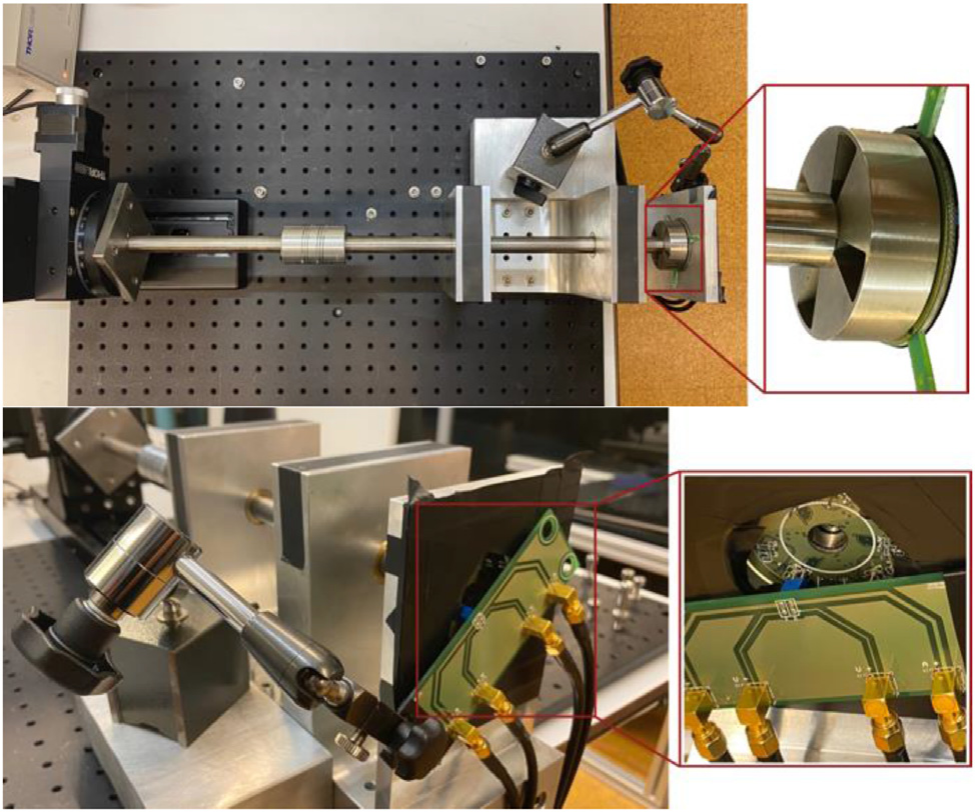
Sensor's setup measurement.

Additionally, to compare the performance of both calculation methods, experimental measurements were performed. The measurement setup used is shown in Figure 12. This setup includes two motorized actuators, one linear (Thorlabs LTS300/M) and one angular (Thorlabs NR360 S/M). They are responsible for positioning the target at the intended position (distance and angle) in relation to the sensor coils. To control the angular actuator, the Thorlabs BSC201 controller was used. To measure the coil inductance, a LCR meter (Keysight E4980AL) was used at 1MHz of frequency.

## 5 Results and discussions

To compare the performance between the two approaches, FEM and the proposed tool, with the experimental measurements, simulations and tests were carried out under the same conditions. The target was positioned at 1 mm of a distance from the sensor. FEM simulations and the proposed tool calculations were done for a target rotation from 0° to 90°, with a step of 5°.

The inductance measurements were performed at each 0.1° rotation of the target, from 0° to 360°. It was considered the full rotation range, and not just 90° as before, to mitigate possible asymmetries that may result from the manufacturing tolerances of the sensor PCB but also from the target mechanics (tilt, offset, etc.). In order to have the most correct measurement, for each position 100 samples were acquired by the LCR meter, and the median was calculated from these. In addition, two sets of these measurements were performed for an inner pair of coils and for an outer. The maximum deviation of measurements for the inner pair is 0.016 nH (0.0019%) and 0.04 nH (0.0034%) for the outer pair. The mean values of inductance obtained experimentally for the two pairs of inner and outer coils are 888.66 nH and 1218.18 nH respectively.

**Figure 13:**
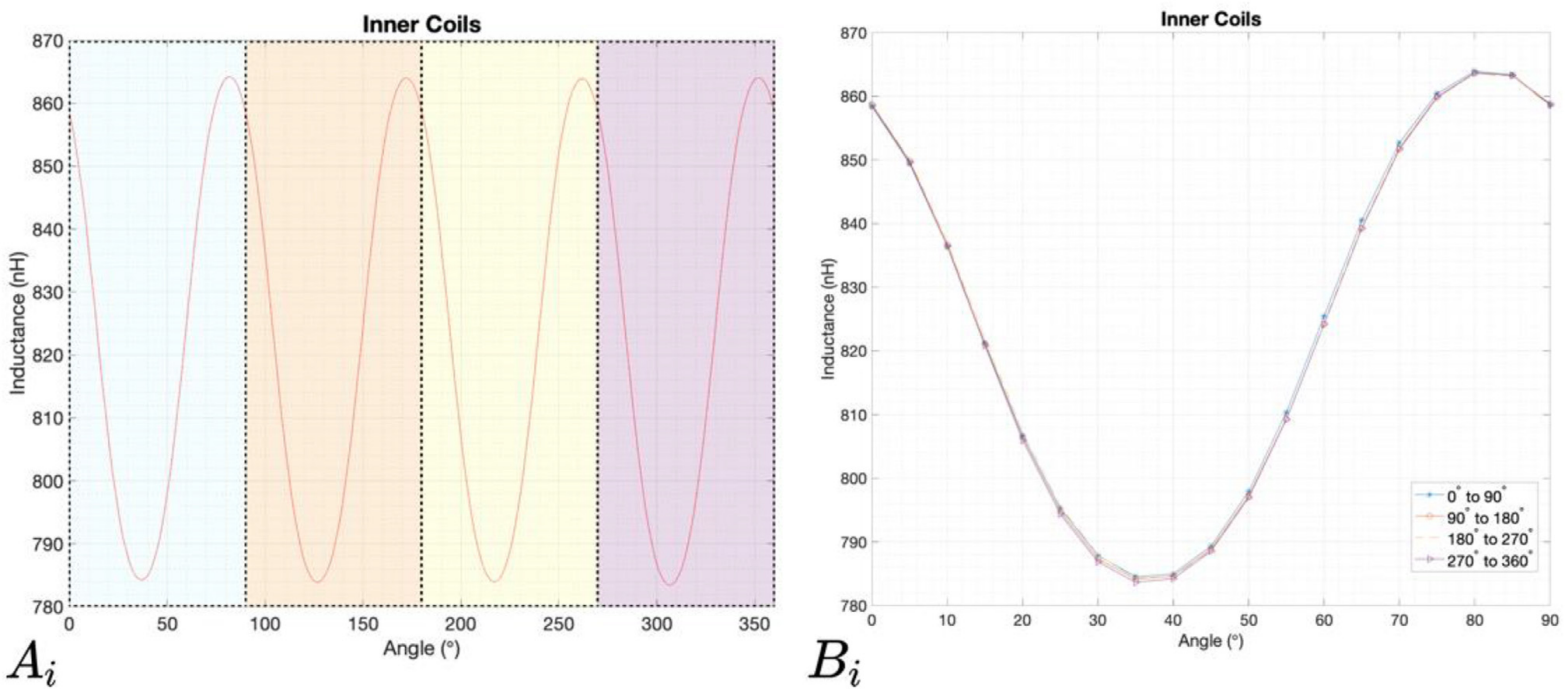
Results of the measured inductance values of an inner pair of coils. $A_{i}$ represents the wave signal resultant from the rotation of the target from 0° to 360°; $B_{i}$ represents the four periods of 90° that exist in a complete rotation overlapped.

**Figure 14:**
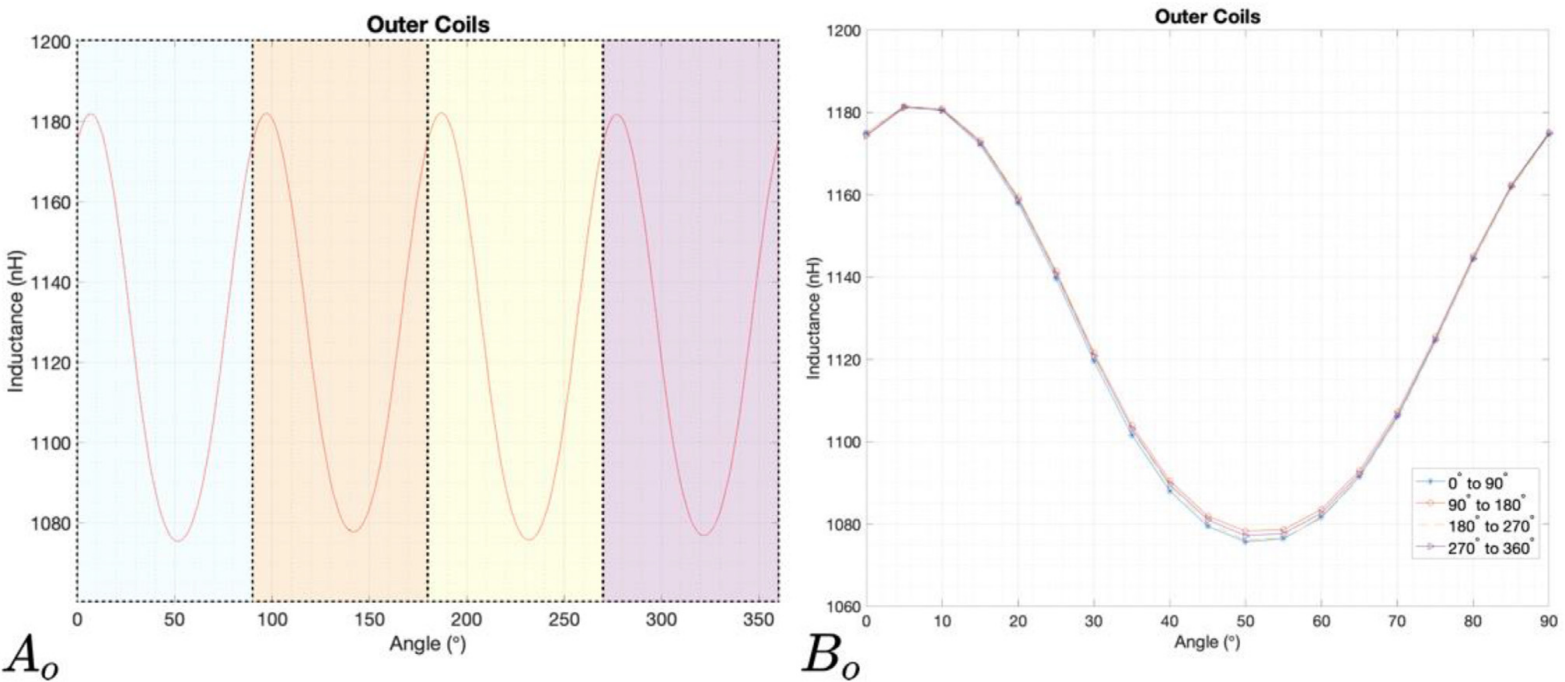
Results of the measured inductance values of an outer pair of coils. A_o_ represents the wave signal resultant from the rotation of the target from 0° to 360°; B_o_ represents the four periods of 90° that exist in a complete rotation overlapped.

Figures 13 and 14 show the results of the measurements for the rotation of the target from 0° to 360°. In Figure 13 $A_{i}$ and Figure 13 $A_{o}$ is visible the wave signal resultant from the rotation of the target from 0° to 360°, and in Figure 14 $B_{i}$ and Figure 14 $B_{o}$ show the four periods of 90° during one full rotation.

It is possible to see, especially in $B_{i}$ and $B_{o}$, that the peaks do not have exactly the same amplitude and that the wave shape has some deviations. In addition to production and mechanical tolerances, these differences may be due to a tilt of the target that was detected in the measurement setup. This has a value of 0.187°, and is reflected in the distance between it and the sensor, ranging from 1 mm to 0.85 mm.

**Figure 15:**
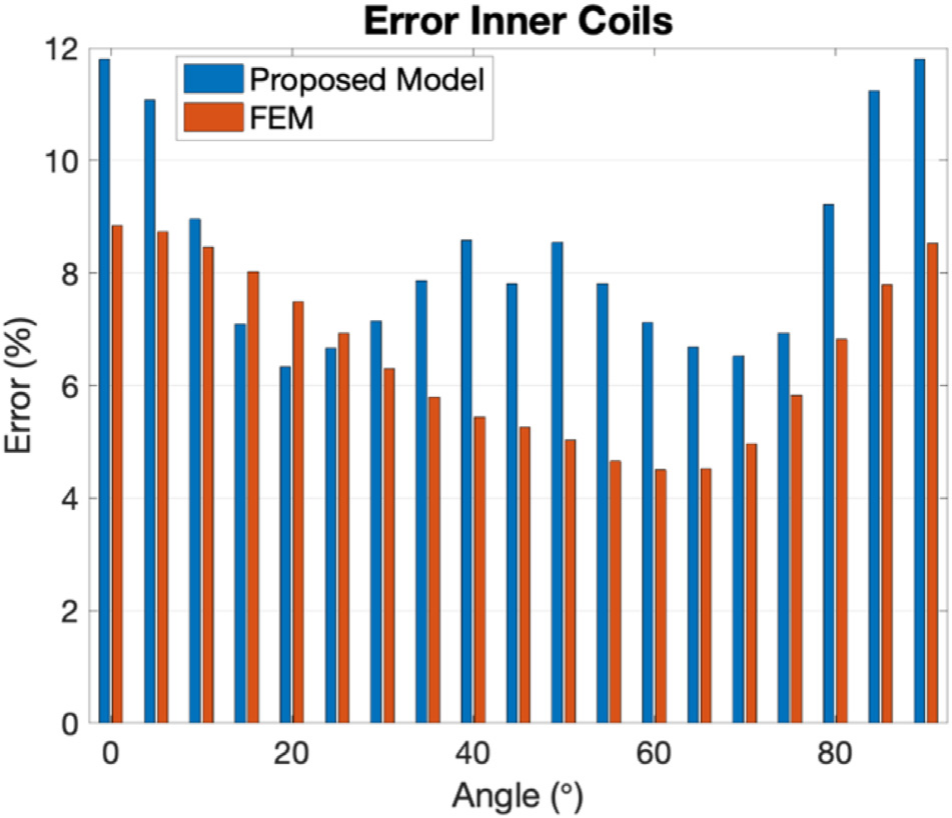
Error between the experimental inductance values and the proposed model (at blue) and the FEM simulation (at orange), for the inner coils case.

**Figure 16:**
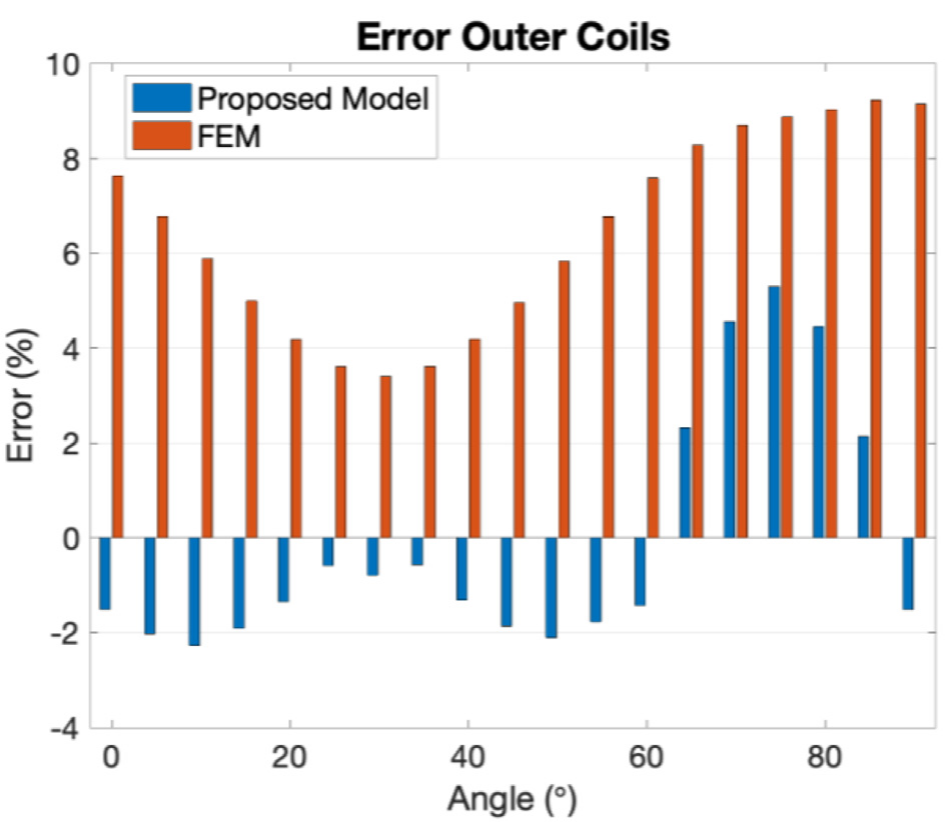
Error between the experimental inductance values and the proposed model (at blue) and the FEM simulation (at orange), for the outer coils case.

Figures 15 and 16 show the errors between the measured inductance values and the calculations from both the developed model and the FEM simulations. It is visible that for the case of internal coils, the errors between the experimental values and the proposed model are slightly higher than the ones from the FEM model. The average error for a 90° target rotation has been calculated to be of 8.38%, and in the case of the FEM is 6.52%. However, the same does not happen in the case of external coils. In this case, the proposed model is more accurate than the FEM, with the first showing an average error of 2.09% and the second of 6.46%.

Comparing the performance of this FEM model with the developed analytical model, it can be seen that the proposed model meets the needs of the research and validation process, reaching similar calculation errors to the more complicated and time consuming FEM simulation. To have the sensor response for a target rotation from 0° to 90° with a step of 5°, the FEM model needs 2 h and 45 min (calculating two tasks simultaneously in a server with two processors Intel(R) Xenon(R) Gold 6130 CPU @2.10 GHz, and a RAM of 128 GB) and the developed model around 5 min (in a MacBook Pro with the processor Intel Core i7 2.6 GHz and 16 GB of memory).

## 6 Conclusions

An analytical versatile tool to estimate the inductance of planar coils in the presence of a conductive target is proposed and validated in this paper. This tool compared to the analytical methods currently used in the literature is faster, more versatile, requires less computational resources, and presents better results. It was validated by comparison with FEM simulations, and experimental measurements on an angular sensor based on eddy currents and planar coils. Taking into account the small difference that exists between the errors obtained by the developed model and FEM simulations (always below 4%), and the great difference that exists in terms of computational resources and calculation time, it can be concluded that the developed model is an asset.

This tool can be further explored to improve its calculations for coils where the current eddy density deviates from the geometric approximation used. This means approximating the target approach to a one-turn coil. It is possible to improve the approximation made to the width of the one-turn coil through a correction factor that takes into account the internal and external diameters of the coil of the sensor. Or considering the target a coil with more than one turn, using a medium width. Therefore, improving this approximation by considering the distribution of these currents would be a good solution.

## Declarations

### Author contribution statement

Andreia Faria: Conceived and designed the experiments; Performed the experiments; Analyzed and interpreted the data; Wrote the paper.

Luis Marques: Conceived and designed the experiments; Analyzed and interpreted the data.

Luis Vale: Performed the experiments; Wrote the paper.

Carlos Ferreira: Contributed reagents, materials, analysis tools or data.

Filipe Alves: Analyzed and interpreted the data; Wrote the paper.

Jorge Cabral: Conceived and designed the experiments; Performed the experiments; Contributed reagents, materials, analysis tools or data.

### Funding statement

Mrs Andreia Faria was supported by Fundação para a Ciência e a Tecnologia [R∖&D Units Project Scope: UIDB/00319/2020 & Grant: PD/BD/128142/2016].

Carlos Ferreira was supported by Fundação para a Ciência e a Tecnologia [Grant: ​PD/BDE/135102/2017].

### Data availability statement

Data included in article/supp. material/referenced in article.

### Declaration of interest’s statement

The authors declare no conflict of interest.

### Additional information

No additional information is available for this paper.
